# Ligand-free preparation of polymer/CuInS_2_ nanocrystal films and the influence of 1,3-benzenedithiol on their photovoltaic performance and charge recombination properties†
†Electronic supplementary information (ESI) available: Additional experimental data (ToF-SIMS results, absorption spectra, EQE spectra, stability and TPC data). See DOI: 10.1039/c8tc05103h


**DOI:** 10.1039/c8tc05103h

**Published:** 2018-12-18

**Authors:** Thomas Rath, Dorothea Scheunemann, Roberto Canteri, Heinz Amenitsch, Jasmin Handl, Karin Wewerka, Gerald Kothleitner, Simon Leimgruber, Astrid-Caroline Knall, Saif A. Haque

**Affiliations:** a Institute for Chemistry and Technology of Materials (ICTM) , NAWI Graz , Graz University of Technology , Stremayrgasse 9 , 8010 Graz , Austria . Email: thomas.rath@tugraz.at; b Energy and Semiconductor Research Laboratory , Department of Physics , Carl von Ossietzky University of Oldenburg , Carl-von-Ossietzky-Strasse 9–11 , 26129 Oldenburg , Germany; c Fondazione Bruno Kessler – Center for Materials and Microsystems , Via Sommarive 18 , I-38123 Povo (Trento) , Italy; d Institute for Inorganic Chemistry , NAWI Graz , Graz University of Technology , Stremayrgasse 9 , 8010 Graz , Austria; e Institute for Electron Microscopy and Nanoanalysis and Center for Electron Microscopy , Graz University of Technology , NAWI Graz , Steyrergasse 17 , 8010 Graz , Austria; f Department of Chemistry and Centre for Plastic Electronics , Imperial College London , Imperial College Road , London , SW7 2AZ , UK

## Abstract

Bulk heterojunction solar cells based on conjugated polymer donors and fullerene-derivative acceptors have received much attention in the last decade. Alternative acceptors like organic non-fullerene acceptors or inorganic nanocrystals have been investigated to a lesser extent; however, they also show great potential. In this study, one focus is set on the investigation of the *in situ* growth of copper indium sulfide nanocrystals in a conjugated polymer matrix. This preparation method allows the fabrication of a hybrid active layer without long-chain ligands, which could hinder charge separation and transport. In contrast, surfactants for the passivation of the nanocrystal surface are missing. To tackle this problem, we modified the absorber layer with 1,3-benzenedithiol and investigated the influence on charge transfer and solar cell performance. Using ToF-SIMS measurements, we could show that 1,3-benzenedithiol is successfully incorporated and homogeneously distributed in the absorber layer, which significantly increases the power conversion efficiency of the corresponding solar cells. This can be correlated to an improved charge transfer between the nanocrystals and the conjugated polymer as revealed by transient absorption spectroscopy as well as prolonged carrier lifetimes as disclosed by transient photovoltage measurements.

## Abstract 2

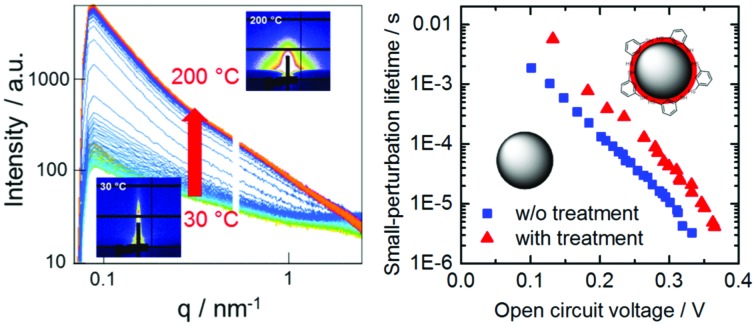
Modification of ligand-free polymer/CuInS_2_ absorber layers led to improved solar cell performance and charge carrier lifetimes.

## 1. Introduction

Non-fullerene acceptors have become a major research topic in the organic solar cell community over the past few years. Besides organic non-fullerene acceptors, which have experienced an impressive increase in efficiency since 2013, inorganic nanocrystals are further promising acceptors in bulk heterojunction solar cells based on conjugated polymers. Inorganic nanocrystals were introduced as acceptors in solar cells by Alivisatos *et al.*^1^ in the year 2002 and due to the combination of organic and inorganic components, the term hybrid solar cells became common for this type of solar cell.

In the following years, continuous research work augmented the understanding of the synthesis, characterization and device assembly of hybrid solar cells and a huge number of possible combinations of inorganic semiconductors and organic conjugated polymers were studied.^2^–^6^ CuInS_2_ nanocrystals are particularly interesting for application in hybrid solar cells due to a well suited optical band gap (1.45 eV) and a high absorption coefficient,^7^ and several studies on their application in hybrid solar cells have been reported.^8^–^16^ The most efficient polymer/nanocrystal hybrid solar cells so far were obtained with PbX (X = S and Se) or CdTe nanoparticles and exhibited power conversion efficiencies (PCEs) between 5 and 6%.^17^,^18^

Hybrid solar cells aim to benefit from both the specific advantages of organic semiconductors, like easy processability and high absorption coefficients, and the high charge carrier mobilities and robustness of inorganic semiconductors. The combination of these two material classes is conceptually very promising; however, especially the interface between organic and inorganic materials is one of the most challenging issues for researchers in this field. The first difficulty occurs in the preparation of a bulk heterojunction absorber layer. Processing inorganic and organic materials in one step from the same solvent can be problematic due to the inherently different chemical and physical properties of both components. This problem is, in the classical approach, tackled by the use of organic capping ligands for the nanocrystals, which facilitate dissolving both components at sufficiently high concentrations to obtain solutions for the coating of the bulk heterojunction layers. However, these capping ligands typically bear long chain alkyl moieties, which also inhibit charge separation at the interface to the conjugated polymer and inter-nanoparticle transport by insulation. Therefore, typically, ligand exchange processes toward shorter ligands are applied to reach high power conversion efficiencies.^2^–^5^

**Fig. 1:**
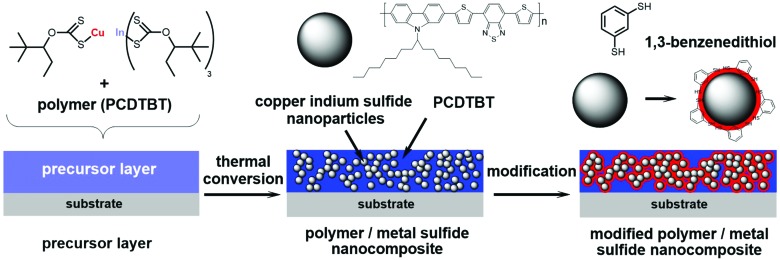
*In situ* formation of the polymer/CuInS_2_ nanocrystal absorber layer using metal xanthates as precursors and the modification with 1,3-benzenedithiol.

An alternative approach to synthesize polymer/nanocrystal hybrid absorber layers without the need for capping ligands was introduced in 2010.^19^ In this approach, nanocrystals were prepared directly in the matrix of the conjugated polymer *via* thermal conversion of metal xanthates to metal sulfide nanocrystals.^15^,^19^,^20^ This facile route leads to ligand-free nanocomposite layers, with PCEs up to 2.8% for CuInS_2_ nanocrystals in combination with a low band gap polymer.^15^Fig. 1 illustrates this *in situ* approach using metal xanthates: in the first step, metal xanthate precursors are dissolved together with a conjugated polymer in an apolar organic solvent. The solution is then coated onto a substrate and the dried layer is subjected to a mild thermal annealing step with temperatures of about 140–200 °C, which is compatible with roll to roll processing on flexible substrates.^16^,^21^ During this annealing step, the metal xanthates decompose, metal sulfide nanocrystals are formed in the polymer matrix and volatile reaction products (COS, CS_2_, and the corresponding alkene) evolve from the layer, so that the resulting nanocomposite layer is free from any impurities.^15^ The formed nanoparticles have a size of 3–5 nm and form a well distributed network with slight agglomeration in the organic matrix.^22^–^24^

Nanocrystals and nanostructures have complex surface properties,^25^ which, due to the high surface-to-volume ratio,^26^ tremendously influence the charge separation and transport dynamics in the corresponding hybrid solar cells.^27^–^29^ In particular, charge carrier trapping, caused by the existence of non-passivated surface states, often plays a more important role in nanocrystal-based systems compared to the situation in polymer/fullerene blends.

In the *in situ* prepared absorber layers, ligands for the passivation of the nanocrystal surfaces are missing, however, introduction of small organic molecules into the *in situ* prepared absorber layer, subsequently after the fabrication process, suggested itself as a potential route for interface modification. For polymer/nanocrystal layers prepared *via* the classical approach using capping ligands, several molecules, such as amines or thiols, have already been introduced into a solid state ligand exchange process to manage surface traps within the absorber layers or to improve the electronic coupling between the polymer and the nanoparticle phase.^30^–^32^

Encouraged by these studies, we investigated how the modification of *in situ* prepared polymer/CuInS_2_ nanocrystal absorber layers with 1,3-benzenedithiol influences charge separation, photovoltaic performance and charge recombination dynamics.

## 2. Experimental procedures

### 2.1. Sample and solar cell preparation

#### Materials

Copper and indium xanthates (copper *O*-2,2-dimethylpentan-3-yl dithiocarbonate and indium *O*-2,2-dimethylpentan-3-yl dithiocarbonate) were synthesized by Aglycon GmbH based on a published protocol^15^ and recrystallized from chloroform/methanol. PCDTBT (poly[[9-(1-octylnonyl)-9*H*-carbazole-2,7-diyl]-2,5-thiophenediyl-2,1,3-benzothiadiazole-4,7-diyl-2,5-thiophenediyl]) was obtained from 1-material. PEDOT:PSS (poly(3,4-ethylenedioxythiophene)–poly(styrenesulfonate)) CLEVIOS™ P VP AI 4083 was obtained from Heraeus. ITO-coated glass slides (15 Ω sq^–1^) were obtained from Lumtec and silver (pellets, 99.99%) was purchased from Kurt J. Lesker Company.

#### Preparation of polymer/CuInS_2_ nanocomposite films

For the preparation of the PCDTBT/CuInS_2_ nanocrystal layers, a precursor solution consisting of 5 mg mL^–1^ PCDTBT, copper xanthate (32.2 mg mL^–1^, 1 equiv.) and indium xanthate (147.8 mg mL^–1^, 1.7 equiv.) dissolved in chlorobenzene was deposited on the respective substrates by spin coating. Thermal conversion was carried out on a hot plate at 195 °C for 15 min (15 min heating time from room temperature to 195 °C) in a nitrogen filled glovebox. The resulting layer thicknesses were between 60 and 90 nm. The layer thicknesses were measured using a Bruker Dektak XT surface profiler.

For modification with 1,3-benzenedithiol, 60 μL of a solution of 1,3-benzenedithiol (5 wt%) in acetonitrile was added dropwise onto the PCDTBT/CuInS_2_ nanocrystal layers. After one minute of soaking time, the solution was spun off (4000 rpm per 20 s). Afterwards, the substrate was washed by adding 60 μL of acetonitrile dropwise onto the substrate followed by spinning off the solvent after 10 s (4000 rpm per 20 s).

For transient absorption spectroscopy (TAS) characterisations, a mesoporous titanium dioxide thin film (prepared by spin coating a 30 NRD Dyesol TiO_2_ nanoparticle paste followed by annealing at 500 °C for 60 min) was coated with a nanocrystalline CuInS_2_ film by spin coating of a precursor solution containing copper and indium xanthates followed by subsequent annealing at 195 °C under inert conditions. Afterwards, the surface of the nanocrystalline CuInS_2_ film was modified with 1,3-benzenedithiol as described above followed by spin coating of the conjugated polymer PCDTBT.

#### Solar cell preparation

ITO-coated glass slides were rinsed with acetone followed by a cleaning step in isopropanol in an ultrasonic bath and an O_2_ plasma etching step (FEMTO, Diener Electronic, Germany). Next, the PEDOT:PSS hole extraction layer was spin coated (2500 rpm, 30 s) on the substrates and subsequently heated to 150 °C for 10 min in a nitrogen filled glove box (resulting layer thickness: 30 nm). The aqueous PEDOT:PSS suspension was filtered through a 0.45 μm PVDF filter (Chromafil Xtra) before spin coating. The PCDTBT/CuInS_2_ nanocrystal absorber layer (60–90 nm, with or without 1,3-benzenedithiol treatment) was prepared as described above directly on the PEDOT:PSS films. Finally, silver cathodes (100 nm) were deposited in a vacuum chamber (8 × 10^–6^ to 1 × 10^–5^ mbar) *via* thermal evaporation through a shadow mask. The active area of the devices was 0.09 cm^2^.

### 2.2. Characterisation techniques

2D grazing incidence small and wide angle X-ray scattering (GISAXS, GIWAXS) measurements were performed at the Austrian SAXS Beamline 5.2L of the electron storage ring ELETTRA (Italy).^33^ The beamline, set to an X-ray energy of 8 keV, was adjusted to a *q*-resolution (*q* = 4π/*λ* × sin(2*θ*/2), where 2*θ* represents the scattering angle) between 0.1 and 3.5 nm^–1^ (GISAXS). The nanocomposite samples were placed in a heating cell (DHS 1100 from Anton Paar GmbH, Graz, Austria) equipped with a custom-made dome with Kapton polyimide film windows and were heated from 30 °C up to 200 °C at a heating rate of approx. 10 °C min^–1^ under a nitrogen atmosphere. During the temperature scan, data were recorded with 6 s time resolution using a Pilatus 1M detector (Dectris). For detection of the GIWAXS signal, a Pilatus 100K detector (Dectris) was used. Angular calibration of the detectors was carried out using silver behenate powder (*d*-spacing of 58.38 Å) and p-bromo benzoic acid.

The invariant of the SAXS data was calculated according to eqn (1) (*q*_*v*_: scattering vector at the cut in nm^–1^, *I*(*q*): scattering intensity, integration regime from *q*_*v*1_ (0.1 nm^–1^) to *q*_*v*2_ (1.3 nm^–1^)):1
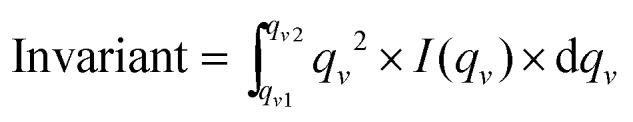



The invariant is very sensitive to structural changes in the sample and describes the integral scattering intensity of the system. It shows the total amount of the scattering material regardless of the structure of the scatterer and depends on the volume fraction of the different phases and the scattering length density contrast.^34^,^35^

ToF-SIMS measurements were performed using a ToF-SIMS IV reflectron time-of-flight secondary ion mass spectrometer (IONTOF GmbH, Muenster, Germany) equipped with a bismuth liquid metal ion gun (LMIG). For each sample, three positive ion spectra and three negative ion spectra were acquired over an area of 150 μm × 150 μm by using Bi_3_^+^ as primary ions, whose dose was maintained below 10^12^ ions × cm^–2^ to ensure static SIMS conditions. The depth profiles were obtained using a sputtering beam of Xe^+^ at 1200 eV of electron impact to sputter a 500 μm × 500 μm rastered area. After 20 s of sputtering, a full spectrum was acquired. The cycle made by 20 s of sputtering and the acquisition of spectra was repeated until the interface with the substrate was reached. The ToF-SIMS spectral data were processed using multivariate analysis (MVA) and, in particular, the Principal Components Analysis (PCA) method was applied. With this aim, the R language and environment were used.^36^

Before carrying out multivariate analysis, the peaks present in each spectrum were integrated, corrected for dead time effects of the registration system, normalized to the primary ion fluencies and mass calibrated. A list of 255 entries, also called features, was then created, gathering the *m*/*z* values of all the integrated peaks from all spectra. This list allowed building a spectral matrix consisting of 6 rows (3 for each sample) and 255 columns, to be used in the subsequent PCA multivariate analysis.

Microsecond transient absorption spectroscopy (μs-TAS) measurements were performed by exciting the samples in an inert atmosphere using a dye laser (Photon Technology International Inc. GL-301) pumped by a nitrogen laser (Photon Technology International Inc. GL-3300). A 100 W quartz halogen lamp (Bentham, IL 1) with a stabilized power supply (Bentham, 605) was used as a probe light source. A silicon photodiode (Hamamatsu Photonics, S1722-01) was used to detect the probe light passing through the sample and the signal was amplified before being passed through electronic band-pass filters (Costronics Electronics). The amplified signal was collected using a digital oscilloscope (Tektronics, DPO3012), which was synchronized with a trigger signal from the pump laser pulse from a photodiode (Thorlabs Inc., DET210).

Transmission electron microscopy (TEM) was performed using a FEI Tecnai T12 electron microscope at an acceleration voltage of 120 kV. Images were taken using a scintillator-based CCD camera, optimized for sensitivity at a low dose (Gatan BioScan Model 792). The samples for TEM measurements were prepared on sodium chloride crystals as a water soluble sacrificial substrate. The free floating films were then transferred to copper TEM grids.

*JV* curves of the solar cells were recorded in a glovebox using a Keithley 2400 source measure unit and custom made LabView software. The samples were illuminated with a Dedolight metal halide lamp with a spectrum similar to the AM 1.5G spectrum at 100 mW cm^–2^. The intensity was calibrated using a Fraunhofer silicon reference solar cell.

Transient photocurrent (TPC) and transient photovoltage (TPV) measurements. TPC and TPV measurements were carried out using a 4 W Seoul P4 light-emitting diode (LED, wavelength 625 nm), driven by a two-channel pulse generator (Agilent 81150A). Continuous white light background illumination was provided from a 4 W white LED (Seoul P4; wavelength 400–700 nm). Transient signals were recorded with a 1 GHz digital storage oscilloscope (Tektronix DPO7104), using an input impedance of either 50 Ω (TPC) or 1 MΩ (TPV). The second output channel of the pulse generator (operating in DC mode) was used to apply an external bias for voltage-dependent TPC measurements. A biased Si photodetector (Thorlabs DET36A) was included in the setup to monitor the switching dynamics of the pulse LED, as well as the relative intensity of the background illumination. The intensity in fractions of suns was estimated by comparing the photocurrent generated from the white light LED with that obtained under simulated AM 1.5G illumination.

## 3. Results and discussion

The PCDTBT/CuInS_2_ nanocomposite films were prepared as described in the introduction and the experimental part. The formed CuInS_2_ nanocrystals have a chalcopyrite crystal structure and sizes between 3 and 5 nm. Detailed properties of the polymer/CuInS_2_ absorber layers can be found in previous publications.^15^,^16^,^22^ In this work, we initially focused on the investigation of the nanocomposite formation by conversion of the metal xanthates to the CuInS_2_ nanocrystals in the PCDTBT matrix. Therefore, we performed simultaneous time resolved grazing incidence small and wide angle X-ray scattering (GISAXS and GIWAXS) measurements with synchrotron radiation. Thin films of the polymer/metal xanthate blend prepared by spin coating on glass substrates were measured during heating from room temperature to 200 °C in a nitrogen atmosphere at a heating rate of 10 °C min^–1^.

**Fig. 2:**
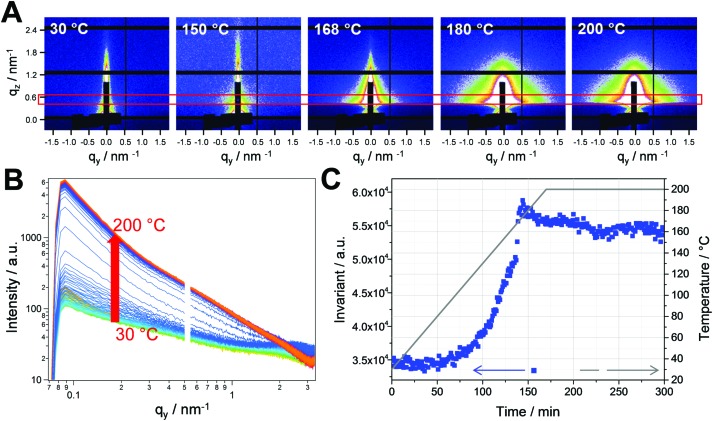
(A) GISAXS images of the sample at different temperatures during the formation process of the PCDTBT/CuInS_2_ nanocomposite thin film, (B) evolution of the in-plane scattering signal and (C) the invariant (blue squares) of the GISAXS curves. The grey line represents the sample temperature during the heating run.

The GISAXS images obtained at selected temperatures during the heating run are depicted in Fig. 2A. The images reveal a significant increase of scattering at higher temperatures in the heating run due to the conversion of the metal xanthates to CuInS_2_ nanocrystals. To further analyse the data, horizontal cuts were calculated by horizontal integration at *q*_*z*_ = 0.5 nm^–1^. The areas used for the integration are marked with a red box in the GISAXS images and the resulting cuts are presented in Fig. 2B. The invariant of the GISAXS curves (shown in Fig. 2C) reveals the first structural changes between 120 and 140 °C. In this temperature range, the decomposition of the mixed copper and indium xanthates also takes place.^15^ The resulting increase of the invariant is continued up to a temperature of 160 °C, where an abrupt rise in scattering intensity takes place. We ascribe this increase to the abrupt formation of the CuInS_2_ nanocrystals at this temperature, leading to a significant increase in electron density in the film, as, besides the formation of metal sulfide, all the organic moieties of the xanthates also evaporate from the film during the conversion to CuInS_2_ nanocrystals.^15^

**Fig. 3:**
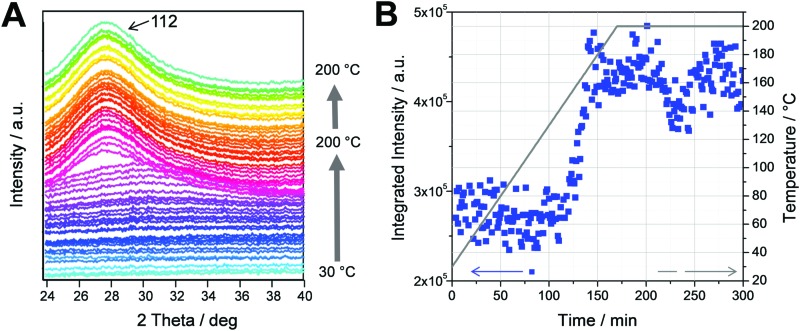
(A) Evolution of the GIWAXS patterns during the heating run from 30 to 200 °C (the patterns are shifted vertically for better visibility) and (B) the integrated intensities (blue squares) of the GIWAXS patterns (integrated from 25.0 to 32.0° 2 theta). The grey line represents the sample temperature during the heating run.

This is supported by the time resolved GIWAXS measurements, which were conducted simultaneously with the GISAXS measurements. The GIWAXS patterns presented in Fig. 3A show the evolution of the (112) reflection of the chalcopyrite structure of the CuInS_2_ nanocrystals at 28° 2*θ*. It can be seen in the graph that the evolution of the (112) reflection occurs very fast from one trace to the other. This is also reflected in the integrated intensities of the GIWAXS curves (calculated between 25.0 and 32.0° 2*θ*), which are plotted *vs.* time in Fig. 3B. The values for the integrated intensities remain constant up to a temperature of 150 °C. Between 150 and 170 °C, a continuous increase is observed and from 170 °C onwards, the values stay constant. This indicates that the crystallization of the nanocrystals takes place between 150 and 170 °C and the growth is completed at 170 °C. The size of the crystallites estimated by the Scherrer equation is approx. 2.1 nm and remains constant after the formation. The primary crystallite size of the CuInS_2_ nanocrystals extracted from the GIWAXS data is slightly smaller than the values extracted from the XRD data in a previous study (2.4 nm),^16^ which can be ascribed to a slight peak broadening due to the grazing incidence setup. Summarizing, the formation process is guided by the first growth of a supramolecular morphology driven by the decomposition of the metal xanthates and the subsequent crystallization of the CuInS_2_ nanocrystals in the temperature range of 150 to 170 °C.

In a next step, we investigated the modification of the PCDTBT/CuInS_2_ nanocomposite layers with 1,3-benzenedithiol to saturate non-passivated surface traps at the CuInS_2_ nanocrystals. Therefore, the films were infiltrated with 1,3-benzenedithiol by dropping a 5% solution of the thiol in acetonitrile on the layers for 60 seconds followed by rinsing the films with pure acetonitrile. If 1,3-benzenedithiol is successfully infiltrated into the nanocomposite film, we assume that it is most likely present at the interface between the nanocrystals and the conjugated polymer (see Fig. 1), since thiols have the tendency to coordinate with the surfaces of metal sulfide nanoparticles.^37^ Moreover, the conjugated polymer phase is rather apolar due to the solubilizing alkyl chains, which makes it unlikely that the polar compound 1,3-benzenedithiol is preferably distributed in the polymer phase.

To confirm the presence of 1,3-benzenedithiol within the nanocomposite layer, time of flight secondary ion mass spectrometry (ToF-SIMS) measurements were performed as ToF-SIMS is a surface analytical technique^38^,^39^ very well known for its excellent chemical specificity and surface sensitivity. The large number of peaks in the ToF-SIMS spectra makes data interpretation and sample comparison a complex task, requiring the application of multivariate analysis techniques, which maximize the usable information contained in the spectra.^40^ In this work, principal component analysis (PCA) was applied to highlight existing variations among the spectra of the non-modified and modified samples and allowed selecting fragments to identify one molecule with respect to the matrix. These fragments were then monitored in further measurements *via* a time profile to understand their in-depth distribution within the 80 nm thick nanocomposite film.

The principal component analysis of the ToF-SIMS positive and negative polarity data captured more than 98% of variance of the data for both polarities. The relationship between the samples (scores plots, see Fig. S1 in the ESI†) shows that the non-modified and the modified samples are completely separated along the first principal component. This means that the PCA completely distinguishes the non-modified sample from the modified sample.

**Fig. 4:**
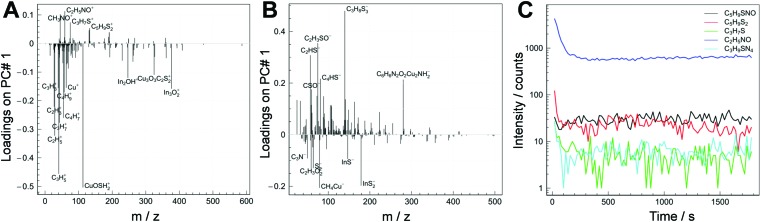
Results of the ToF-SIMS study to prove the infiltration of 1,3-benzenedithiol into the absorber layer. Comparison of the non-modified and the modified sample: loading plots of positive ions (A) and negative ions (B) whereby the positive values are characteristic of the modified sample and the negative values are characteristic of the non-modified sample; (C) depth profile of the respective positive ions in the polymer/CuInS_2_ nanocrystal layer.

In Fig. 4 (A: positive polarity; B: negative polarity), the loading plots of a modified sample and a non-modified sample are depicted. The positive values of loadings in Fig. 4A and B show which ion fragments are characteristic for the modified sample. Along the negative values of loadings are fragments that are characteristic for the non-modified sample. Some characteristic ones are labelled in the figure. The obtained ToF-SIMS data reveal that there is a distinct difference in the chemical composition of the non-modified sample and the modified sample. The molecule fragments representative of the modified sample are mainly organo-sulfur species that are very likely to be fragmentation products of 1,3-benzenedithiol. In contrast to this, typical fragments for the non-modified sample are mainly alkanes and In or Cu containing compounds, which might stem from the conjugated polymer and the nanocrystals.

Furthermore, to study if 1,3-benzenedithiol is homogeneously distributed over the whole depth of the polymer/CuInS_2_ nanocomposite layer, the distribution of the characteristic fragments of the modified sample within the nanocomposite layer was also probed with ToF-SIMS. The depth profiles of the positive fragments are reported in Fig. 4C. The depth profile is shown over the full thickness of the absorber layer up to close to the interface with the substrate. As can be seen in the graph, the fragments characteristic of the 1,3-benzenedithiol modified sample are homogeneously distributed in the film. In the first monolayers of the surface, the signals are probably affected by the presence of contaminants and elements that could enhance the ion yield.^39^ Based on these investigations, it is evident that 1,3-benzenedithiol was successfully incorporated in the nanocomposite layer by the used infiltration process and homogeneously distributed within the whole depth of the film.

**Fig. 5:**
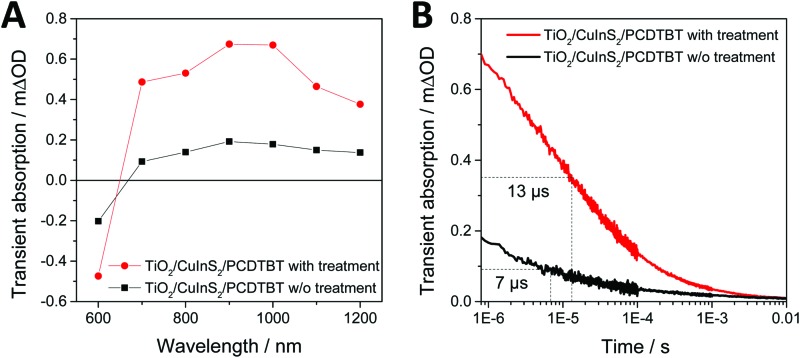
(A) Transient absorption spectra of mesoporous TiO_2_/CuInS_2_/PCDTBT thin films with and without 1,3-benzenedithiol modification measured 1 μs after pulsed laser excitation (wavelength: 450 nm) and (B) the corresponding transient absorption kinetics measured using a probe wavelength of 1000 nm.

Based on the discussion above, it is reasonable to assume that thiol is present (indeed located) at the interface of the conjugated polymer and the nanoparticles due to the high affinity of the –SH groups to the metal ions (Cu^+^ and In^3+^) at the surface of the CuInS_2_ nanocrystals. Consequently, the presence of thiol at the polymer/CuInS_2_ interface may influence interfacial charge transfer at the heterojunction. To investigate this possible effect, we performed transient absorption spectroscopy (TAS) measurements on mp-TiO_2_/CuInS_2_/PCDTBT model samples. These samples were chosen as they enabled us to introduce 1,3-benzenedithiol directly at the CuInS_2_/PCDTBT interface by soaking the mp-TiO_2_/CuInS_2_ films in a solution of 1,3-benzenedithiol in acetonitrile followed by washing the sample with acetonitrile and coating the PCDTBT layer. The TAS spectra of the samples with and without 1,3-benzenedithiol modification are shown in Fig. 5A. In both samples, a broad transient absorption peak around 700 to 1100 nm is observed, which is typical for hole polarons present in the conjugated polymer PCDTBT.^22^,^41^ As both components absorb at 450 nm, the wavelength of the pulsed laser excitation (see Fig. S2, ESI†), it cannot be clearly concluded if the hole polarons formed in the PCDTBT film result from hole transfer from CuInS_2_ to PCDTBT or from electron transfer from the polymer to the nanocrystals. The intensity of the transient absorption peak observed in the modified sample (red trace) is significantly larger than that observed in the non-modified sample. As the intensity of the signals can be directly related to charge generation yields, these measurements clearly demonstrate an enhanced charge transfer across the PCDTBT/CuInS_2_ heterojunction due to the modification with 1,3-benzenedithiol. Furthermore, the kinetics of electron/hole recombination in the polymer/CuInS_2_ heterojunctions were studied by probing the decrease of the PCDTBT polaron band at 1000 nm as a function of time. The observed transient absorption kinetics are plotted in Fig. 5B. The recombination lifetime (*τ*_50%_) was obtained by determining the time after the transient absorption signal had decreased by 50% of its initial value. This analysis reveals that the charge carriers in the modified sample are significantly longer lived compared to the reference sample. For the non-modified sample, a *τ*_50%_ recombination lifetime of approx. 7 μs was found, whereas the lifetime is increased to about 13 μs in the modified sample.

**Fig. 6:**
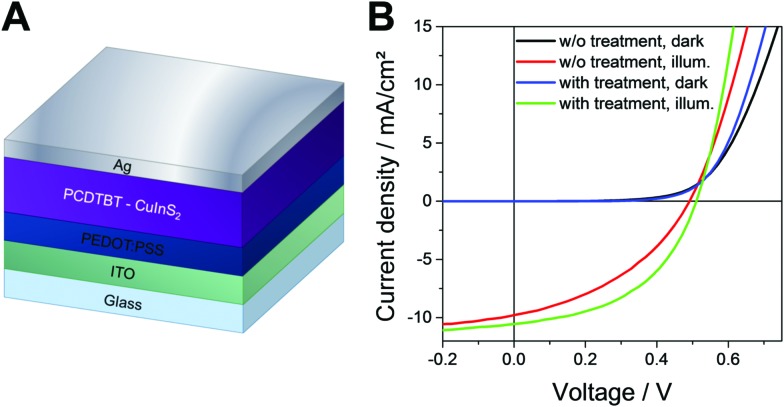
(A) Scheme of the solar cell architecture and (B) *JV* curves of typical PCDTBT/CuInS_2_ solar cells with and without 1,3-benzenedithiol modification.

Next, we investigated the influence of the 1,3-benzenedithiol modification on the solar cell performance. Therefore, hybrid solar cells were prepared with the following device architecture: glass/ITO/PEDOT:PSS/PCDTBT-CuInS_2_/Ag. The *JV* curves measured in the dark and under 100 mW cm^–2^ illumination of typical solar cells with and without 1,3-benzenedithiol modification prepared in this study are shown in Fig. 6B. The characteristic solar cell parameters are summarized in Table 1. It can be seen that the short circuit current density (*J*_SC_) and the open circuit voltage (*V*_OC_) are slightly increased upon the 1,3-benzenedithiol modification. The *V*_OC_ of the solar cells is around 500 mV and the *J*_SC_ was found to increase from 9.5 to 10.3 mA cm^–2^ on average. However, the fill factor (FF) is notably increased by the modification. The average fill factors of the devices without modification are 0.41 ± 0.04, while for the solar cells with the 1,3-benzenedithiol modified absorber layer, values of 0.49 ± 0.05 could be obtained. Accordingly, the modified devices also showed lower series resistances and higher shunt resistances. The highest PCEs of the solar cells with a modified absorber layer ranged between 2.4 and 2.7% in this study. Without modification, the PCEs of the solar cells were found to be around 1.8–1.9% on average. The EQE spectra of the non-modified and modified solar cells are shown in Fig. S3 (ESI†) and exhibit a similar shape. It is only in the wavelength region around 600 nm, where PCDTBT has its absorption maximum, that the EQE values of the modified solar cell are slightly higher. This suggests that electron transfer from the polymer to the nanocrystal phase is enhanced due to the 1,3-benzenedithiol modification.

**Table 1:** Characteristic solar cell parameters of typical PCDTBT/CuInS_2_ solar cells with and without modification (including average values and standard deviations calculated from the eight best devices each)

	*V*_OC_/V	*J*_SC_/mA cm^–2^	FF	PCE/%	*R*_s_/Ω cm^2^	*R*_sh_/Ω cm^2^
w/o modification	0.490	9.75	0.40	1.87	15	180
	0.487 ± 0.025	9.51 ± 0.41	0.41 ± 0.04	1.87 ± 0.30	—	—

with modification	0.501	10.53	0.48	2.56	11	300
	0.503 ± 0.015	10.27 ± 0.38	0.49 ± 0.05	2.53 ± 0.28	—	—

To confirm these results, we used a second conjugated polymer and studied the influence of 1,3-benzenedithiol on polymer/CuInS_2_ hybrid solar cells. With PPD–BDT as a conjugated polymer, consisting of pyrrolopyridazinedione (PPD) acceptor units and benzodithiophene (BDT) donor units,^42^ very similar results as for PCDTBT are obtained (see Table S1, ESI†). As a consequence of the 1,3-benzenedithiol modification, the *V*_OC_ stays almost constant, the *J*_SC_ is increased from 8.2 to 10.0 mA cm^–2^ and the FF increases from 0.44 to 0.51, which leads to an enhancement of the PCE from 1.8 to 2.5%.

In previous studies, it was found that the long-term stability of ligand-free prepared polymer/CuInS_2_ solar cells is very promising as the PCE of the investigated solar cells stayed almost constant over several hundreds of hours of constant illumination.^16^,^43^ To investigate if 1,3-benzenedithiol has a negative influence on the device stability, we also tested an encapsulated PPD–BDT/CuInS_2_ solar cell under continuous illumination at maximum power point conditions. The results shown in Fig. S4 (ESI†) reveal that 1,3-benzenedithiol has no negative impact on device stability. The characteristic parameters of the solar cell show only a slight decrease over the period of the lifetime test of 2000 h. While the I_SC_ and the FF stay almost constant, the *V*_OC_ slightly decreases, which leads to a loss in PCE of approx. 10% within the lifetime test.

**Fig. 7:**
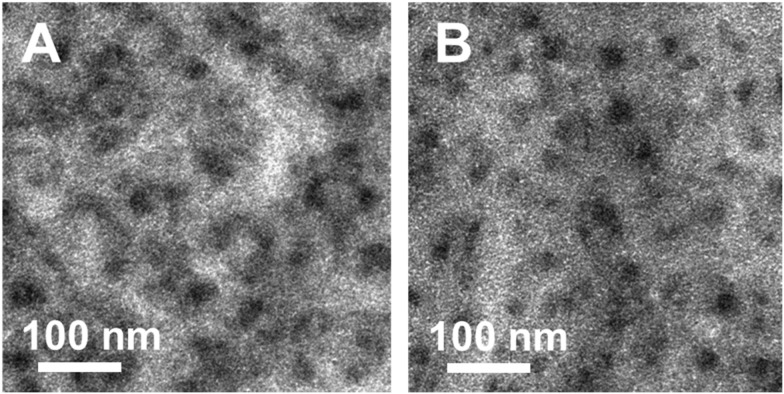
TEM images of PCDTBT/CuInS_2_ absorber layers without (A) and with (B) 1,3-benzenedithiol modification.

Moreover, to investigate if the morphology of the hybrid absorber layer is affected by the 1,3-benzenedithiol modification, TEM images were recorded. These images are shown in Fig. 7 and indicate that the morphology is not altered by the modification step. The darker areas represent the nanocrystal phase consisting of nanocrystals with a typical size of 3–5 nm, which tend to agglomerate.^15^,^16^,^22^ Also, in the brighter areas (indicating the polymer phase), nanocrystals are present, forming a continuous network within the polymer phase.

To further elucidate the influence of the 1,3-benzenedithiol modification on the charge carrier collection and recombination dynamics, transient photocurrent (TPC) and transient photovoltage (TPV) measurements were performed. For TPC measurements, devices were held at a continuous voltage bias and background illumination, while the photocurrent decay *j*(*t*) after square pulse excitation was recorded. For quantitative analysis, we determined the total amount of extracted charge,2
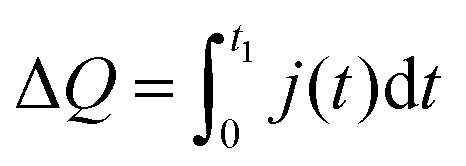
where *t*_1_ is the time at which the transient signal was no longer detectable.

**Fig. 8:**
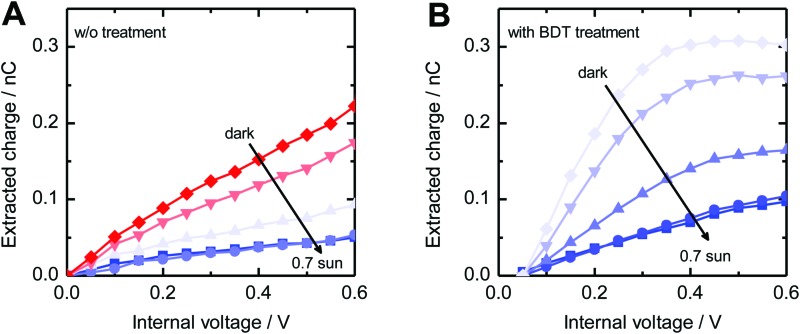
Extracted charge Δ*Q versus* internal voltage *V*_int_ ≈ *V*_OC_ – *V* in dependence of the background illumination intensity (as indicated by arrows) for PCDTBT/CuInS_2_ solar cells (A) without and (B) with 1,3-benzenedithiol modification.

Fig. 8 shows the extracted charge as a function of the background light intensity and internal voltage *V*_int_ ≈ *V*_OC_ – *V*. For all light intensities, the absolute amount of extracted charge increased in the 1,3-benzenedithiol modified sample compared to the reference sample. Moreover, we observed a drastic reduction of Δ*Q* with increasing background light intensity. This decline is directly correlated to the gradual disappearance of the long-lived current tail in the photocurrent decay dynamics (see Fig. S5, ESI†). Such behaviour is commonly attributed to the filling of shallow bulk traps with increasing steady state carrier concentration.^44^,^45^ The decrease of the extracted charge with decreasing internal voltage is associated with recombination competing with charge carrier extraction due to the slow-down of charge collection upon reduction of the internal electric field. Moreover, for *V*_int_ → 0, charge injection from the electrodes becomes significant.

**Fig. 9:**
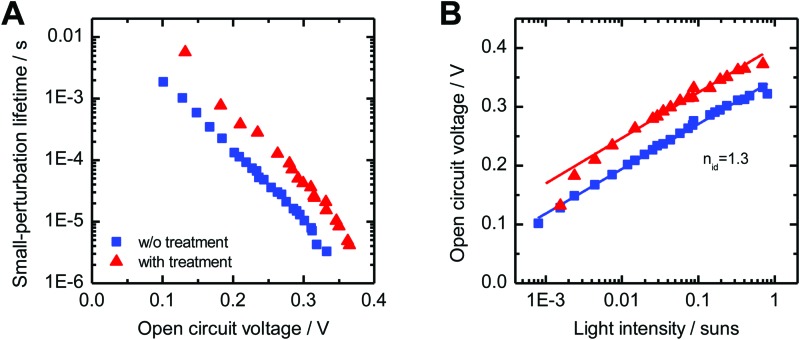
(A) Small-perturbation lifetime *τ*_Δ*n*_ determined from TPV measurements *versus* open circuit voltage and (B) and the light intensity dependence of the open circuit voltage. Lines indicate fits to eqn (3).

While TPC studies provide information about charge collection, we used TPV measurements to study the dynamics of charge recombination. For this purpose, the device is kept at open circuit and under continuous background illumination, while the photovoltage decay induced by a light pulse is recorded. Light intensities were chosen such that the pulse can be considered as a small perturbation. The decay dynamics after switching off the pulse can be described for both sample systems by a mono-exponential decay with the small-perturbation lifetime *τ*_Δ*n*_. Fig. 9A shows the dependence of *τ*_Δ*n*_ on the open circuit voltage. At a fixed open circuit voltage, the modified samples show an up to five times longer small-perturbation lifetime, which is consistent with the trends observed in the μs-TAS measurements.

Fig. 9B shows the intensity dependence of the open circuit voltage. As the recombination and generation rates are balanced at open circuit, and the generation rate is assumed to be proportional to the light intensity *I*, the relationship between *V*_OC_ and *I* is proportional to the ideality factor *n*_id_^46^,^47^
3
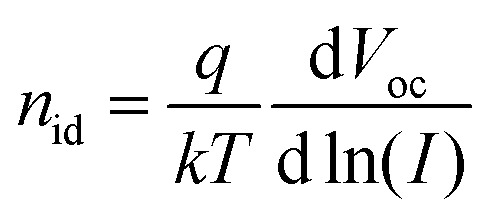
where *q* is the elementary charge, *k* is the Boltzmann constant, and *T* is the temperature. An ideality factor approaching *n*_id_ = 2 has previously been attributed to trap-assisted recombination *via* sub-band gap states,^48^,^49^ while an ideality factor close to 1 is commonly assigned to a direct recombination mechanism such as Langevin recombination. Fitting the data in Fig. 9B with eqn (3) yields an ideality factor of *n*_id_ ≈ 1.3, similar to those of other hybrid blend systems,^50^ while for devices with an active layer consisting solely of colloidal CuInS_2_ nanocrystals, an ideality factor of 2 was observed.^51^ The observed ideality factors indicate that besides bimolecular recombination, a trap-mediated process is also present in the modified as well as the non-modified system.

## 4. Conclusion

In conclusion, this study revealed that the formation of CuInS_2_ nanocrystals from metal xanthates within the conjugated polymer matrix takes place at approx. 160 °C. Slight structural changes in the films due to starting decomposition of the metal xanthates are already observed at lower temperatures; however, the formation of nanocrystals proceeds within a very narrow temperature window. Moreover, we could show for the first time that a post-synthesis modification of *in situ* and ligand-free prepared polymer/CuInS_2_ nanocrystal hybrid layers with 1,3-benzenedithiol leads to enhanced photovoltaic properties of these nanocomposite films most likely due to the saturation of non-passivated surface traps of the CuInS_2_ nanocrystals. The presence of 1,3-benzenedithiol in the polymer/CuInS_2_ absorber layers, which was confirmed by ToF-SIMS measurements, leads to an increase in power conversion efficiency of the corresponding solar cells from 1.9 to 2.5%. This increase is mainly based on a higher *J*_SC_ as well as a higher FF, which matches well with transient absorption spectroscopy data, indicating an enhanced charge carrier generation yield as well as longer charge carrier lifetimes in 1,3-benzenedithiol modified samples. Also, TPC data showed that the absolute amount of extracted charges is increased in the solar cells with the modified absorber layer. Furthermore, the significant change in FF allows us to assume that the 1,3-benzenedithiol treatment has an influence on the recombination properties of the solar cells. The performed TPV measurements revealed significantly slower recombination dynamics (five times longer small-perturbation lifetime) in the modified sample; however, the ideality factor is similar (*n*_id_ ≈ 1.3) in the modified and the non-modified solar cells, which indicates that in addition to bimolecular recombination, trap-mediated recombination also occurs in both samples. The presented post-synthesis modification approach can be extended further to small organic ligand molecules and is a valuable tool for further improvement of the performance of *in situ* prepared polymer/nanocrystal hybrid solar cells.

## Conflicts of interest

There are no conflicts of interest to declare.

## Supplementary Material

Supplementary information
