# Supplementary material for: Ligand-free preparation of polymer/CuInS2 nanocrystal films and the influence of 1,3-benzenedithiol on their photovoltaic performance and charge recombination properties
Source: J Mater Chem C Mater. 2018 Dec 18;7(4):943–52. doi: 10.1039/c8tc05103h (PMC6350655; doi:10.1039/c8tc05103h)
Supplement: Supplementary file 1 [file TC-007-C8TC05103H-s001.pdf]

Electronic Supplementary Material (ESI) for Journal of Materials Chemistry C.
This journal is © The Royal Society of Chemistry 2018

Supporting Information

Ligand-free preparation of polymer/$CuInS_2$ nanocrystal films and the influence of 1,3-benzenedithiol on their photovoltaic performance and charge recombination properties

Thomas Rath,[1,*] Dorothea Scheunemann,[2] Roberto Canteri,[3] Heinz Amenitsch,[4] Jasmin Handl,[1] Karin Wewerka,[5] Gerald Kothleitner,[5] Simon Leimgruber,[1] Astrid-Caroline Knall,[1] and Saif A. Haque[6]

[1] Institute for Chemistry and Technology of Materials (ICTM), NAWI Graz, Graz University of Technology, Stremayrgasse 9, 8010 Graz, Austria

[2] Energy and Semiconductor Research Laboratory, Department of Physics, Carl von Ossietzky University of Oldenburg, Carl-von-Ossietzky-Strasse 9−11, 26129 Oldenburg, Germany

[3] Fondazione Bruno Kessler - Center for Materials and Microsystems, Via Sommarive 18, I-38123 Povo (Trento), Italy

[4] Institute for Inorganic Chemistry, NAWI Graz, Graz University of Technology, Stremayrgasse 9, 8010 Graz, Austria

[5] Institute for Electron Microscopy and Nanoanalysis and Center for Electron Microscopy, Graz University of Technology, NAWI Graz, Steyrergasse 17, 8010 Graz, Austria

[6] Department of Chemistry and Centre for Plastic Electronics, Imperial College London, Imperial College Road, London, SW7 2AZ, UK

* Corresponding author address: Institute for Chemistry and Technology of Materials (ICTM), NAWI Graz, Graz University of Technology, Stremayrgasse 9, 8010 Graz, Austria; Email: thomas.rath@tugraz.at

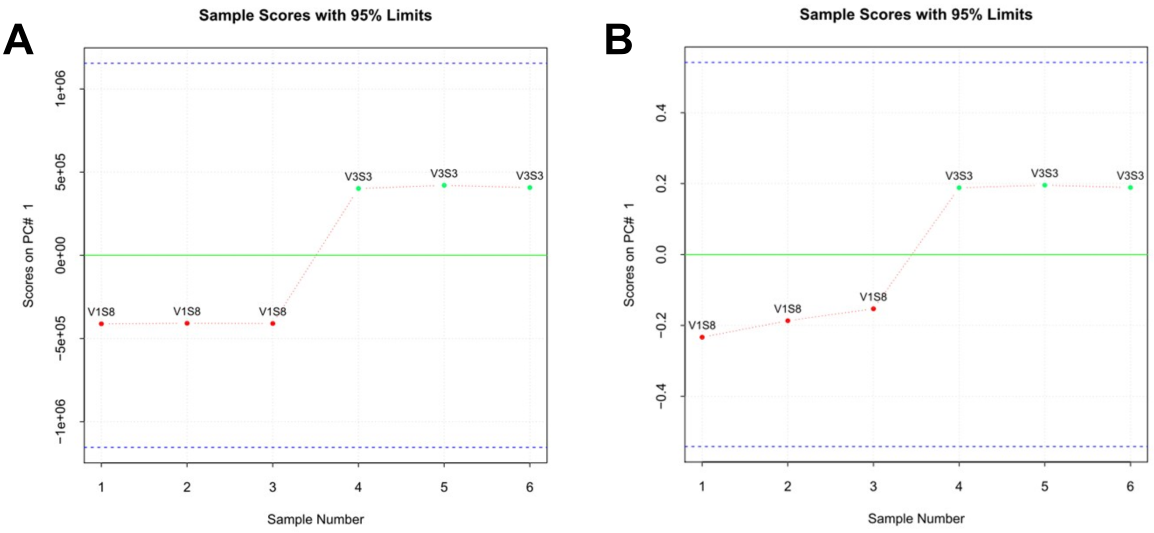

Fig. S1 Scores plots (A: negative ions, B: positive ions) extracted from the ToF-SIMS measurements. The red dots represent the non-modified sample, the green dots the sample modified with 1,3-benzenedithiol.

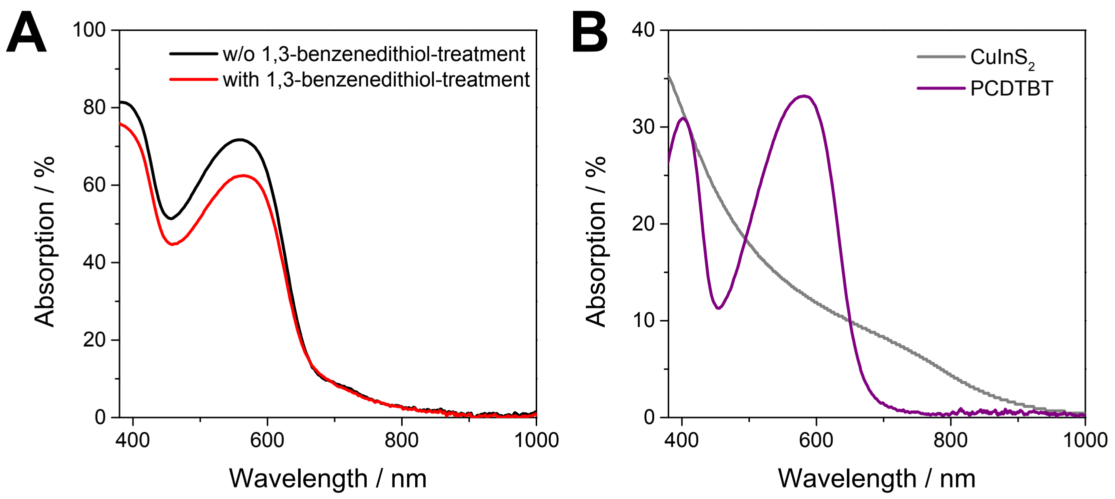

Fig. S2 (A) UV-Vis absorption spectra of the mp-TiO_2/CIS/PCDTBT films on glass (with and without 1,3-benzenedithiol treatment) used for the transient absorption spectroscopy measurements; (B) UV-Vis spectra of pristine PCDTBT and $CuInS_2$ thin films on glass.

For the determination of the absorption spectra, transmission and reflection spectra were recorded on a Shimadzu 2600 spectrophotometer equipped with an ISR-2600Plus integrating sphere attachment.

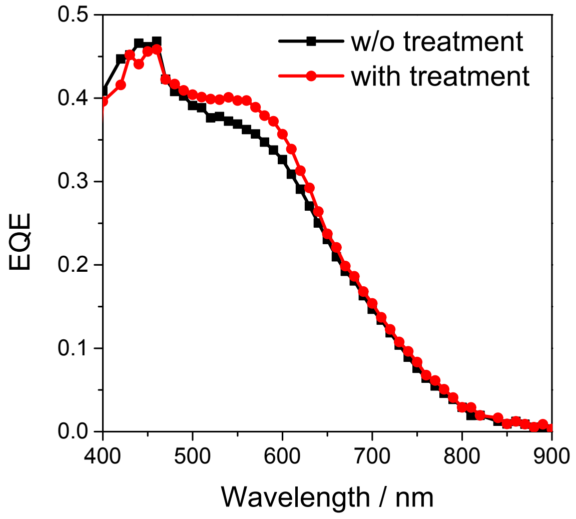

Fig. S3 EQE spectra of PCDTBT/$CuInS_2$ solar cells without and with 1,3-benzenedithiol modification.

The EQE spectra were acquired using a MuLTImode 4 monochromator (Amko) equipped with a 75 W xenon lamp (LPS 210-U, Amko), a lock-in amplifier (Stanford Research Systems, Model SR830), and a Keithley 2400 source meter. The monochromatic light was chopped at a frequency of 30 Hz and the measurement setup was spectrally calibrated with a silicon photodiode (Newport Corporation, 818-UV/DB).

Table S1 Characteristic solar cell parameters of PPD–BDT/$CuInS_2$ solar cells with and without 1,3-benzenedithiol modification (average values and standard deviations are calculated from the five best devices each)

	V_{OC} / V	J_{SC} / mA/cm²	FF	PCE / %
w/o modification	0.498 ± 0.018	8.22 ± 1.02	0.44 ± 0.05	1.81 ± 0.36
with modification	0.494 ± 0.010	10.06 ± 0.23	0.51 ± 0.02	2.54 ± 0.13

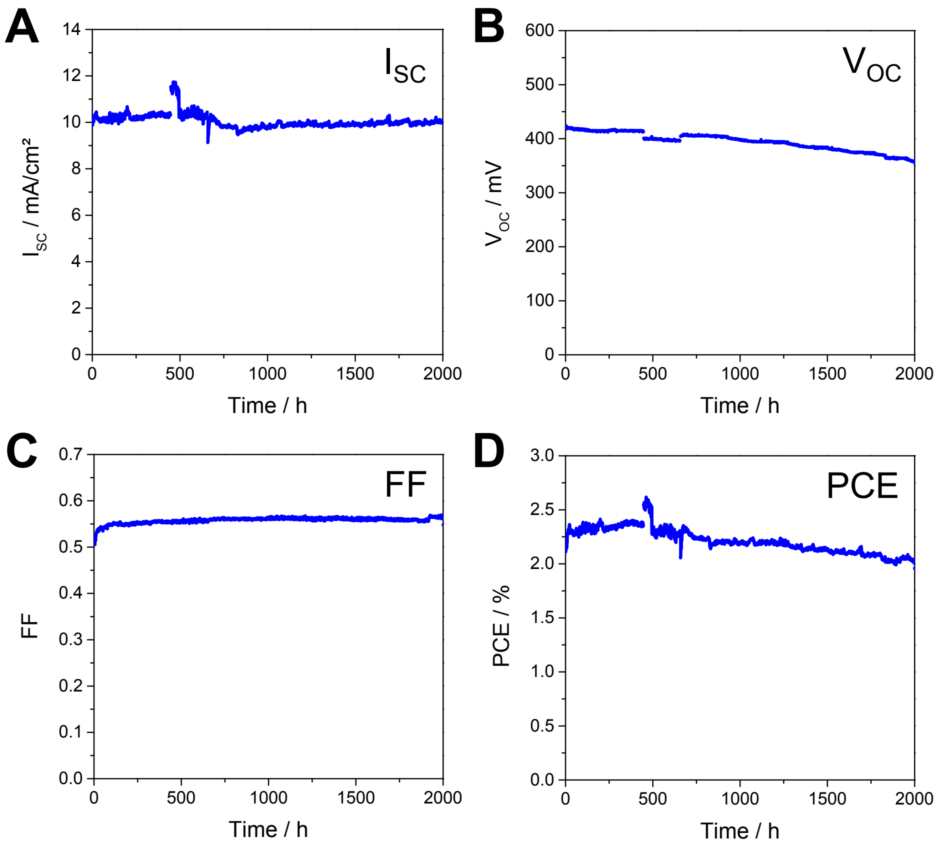

Fig. S4 Results of a 2000 h stability test of a PPD-BDT/$CuInS_2$ solar cell modified with 1,3-benzenedithiol. The slight artefacts present in the characteristic solar cell data around 500 h are due to irregularities of the measuring setup during this time period.

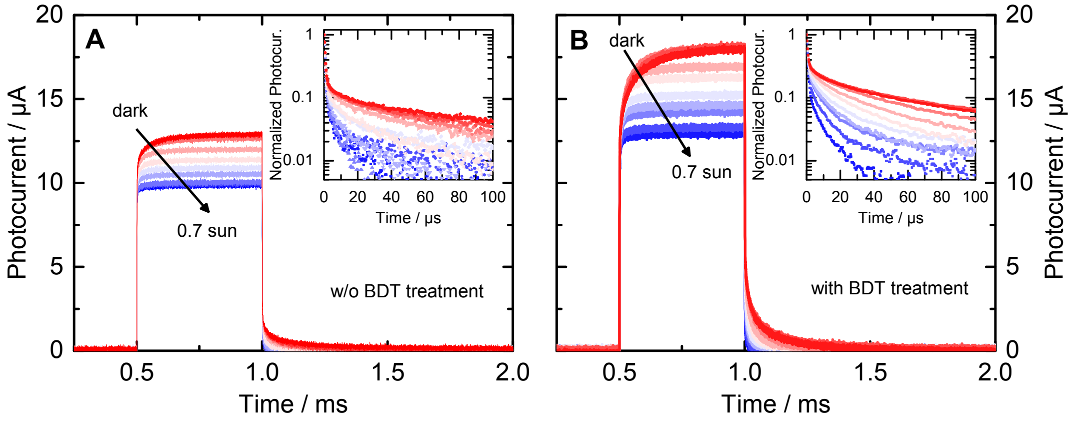

Fig. S5 TPC signals as a function of white-light background illumination intensity (dark to 0.7 suns, zero bias voltage) for PCDTBT/$CuInS_2$ solar cells (A) without and (B) with 1,3-benzenedithiol modification. The insets show the corresponding normalized photocurrent transients.
